# Incidence of Pituitary Apoplexy and Its Risk Factors in Chinese People: A Database Study of Patients with Pituitary Adenoma

**DOI:** 10.1371/journal.pone.0139088

**Published:** 2015-09-25

**Authors:** Xiaoming Zhu, Yongfei Wang, Xuelan Zhao, Cuiping Jiang, Qiongyue Zhang, Wenjuan Jiang, Yan Wang, Haixia Chen, Xuefei Shou, Yao Zhao, Yiming Li, Shiqi Li, Hongying Ye

**Affiliations:** 1 Department of Endocrinology & Metabolism, Huashan Hospital, Shanghai Medical College, Fudan University, Shanghai, China; 2 Department of Endocrinology & Metabolism, Huadong Hospital, Shanghai Medical College, Fudan University, Shanghai, China; 3 Department of Endocrinology & Metabolism, Jinshan Hospital, Shanghai Medical College, Fudan University, Shanghai, China; 4 Department of Neuropathology, Huashan Hospital, Shanghai Medical College, Fudan University, Shanghai, China; 5 Department of Neurosurgery, Huashan Hospital, Shanghai Medical College, Fudan University, Shanghai, China; 6 Shanghai Pituitary Tumor Center, Shanghai, China; Central South University, CHINA

## Abstract

**Background:**

There are few studies of the incidence and clinical characteristics of pituitary apoplexy (PA) in pituitary adenoma patients, and the findings have been inconsistent.

**Objective:**

The aim of the study was to retrospectively assess the incidence, clinical presentation, surgical management and postoperative complications of PA in pituitary adenoma patients.

**Methods:**

A database was specifically designed to collect clinical, therapeutic, prognostic and histological information about pituitary adenoma patients. Using multivariate logistic regression, odds ratios (ORs) with 95% confidence intervals (CIs) were calculated to identify associated factors.

**Results:**

A total of 2021 pituitary adenoma patients were recruited. 97 (4.8%) patients had PA. The incidence of PA was 10.11% in patients with pituitary macroadenoma, and 0.36% in patients with microadenoma. Variables for the logistic regression model independently associated with PA were sex (male vs. female, OR = 2.54, 95% CI: 1.59~4.07), tumor type (negative staining vs. positive staining, OR = 2.04, 95% CI: 1.29~3.23), and tumor size (macroadenoma vs. microadenoma, OR = 26.46, 95% CI = 9.66~72.46). Headache, visual deterioration, and vomiting were the most common symptoms in patients with pituitary adenoma. Patients with and without PA had similar frequency of visual deterioration, head trauma, acromegalic appearance, galactorrhoea, cold intolerance and Cushingoid appearance, but headache, vomiting, ptosis, diplopia, fever and blindness were significantly more common in patients with PA. Pearson Chi-Square tests revealed a significant difference in surgical approach between patients with and without PA (95.88% vs. 85.57%, P = 0.01).

**Conclusion:**

Our findings suggest that PA is not a rare event. Male sex, non-functioning tumor, and macroadenoma are associated with an increased risk of PA. Compared with pituitary adenoma patients without PA, patients with PA have more severe symptoms.

## Introduction

Pituitary apoplexy (PA) is caused by hemorrhage or infarction of a pituitary adenoma. It is characterized by the sudden onset of headache, vomiting, visual impairment and decreased consciousness [1,2,3].

Few studies of PA in pituitary neoplasms have been performed, however the findings of these studies are inconsistent [2,4,5,6,7,8]. There are some factors that contribute to this inconsistency. Firstly, the majority of previous reports of PA are small case series. Secondly, most reports are based on retrospective analyses [6]. There have been some changes in the diagnosis, treatment and imaging of PA. Finally, some studies only included patients with non-functioning pituitary adenomas [4,9] and macroadenoma [10,11]. Characteristics of PA in Chinese populations have rarely been reported. Mou et al [12] described 83 of 426 (19.48%) patients presented with PA. Male patients and patients with functional adenoma had a higher probability of developing apoplexy. Except for PRL-secreting tumors, expression of PCNA in patients with PA was significantly higher than that in patients without PA.

Doctors in China are facing a number of challenges in the management of PA. There are more than one thousand surgical procedures for pituitary tumor every year in our pituitary adenoma center [13,14]. The diagnostic criteria for PA are inconsistent. In most cases, PA occurs in an undiagnosed, preexisting pituitary adenoma. Patients with suspected pituitary apoplexy require detailed visual acuity, visual field, and oculomotor nerve examination, hormone profile assessment, and emergency glucocorticoid treatment. No evidence from randomized studies exists in the management of pituitary apoplexy.

To identify factors associated with PA, we retrospectively analyzed data from 2021 patients with pituitary adenoma. A computerized database was developed in our center to collect all information about clinical, biochemical, radiological, and ophthalmological outcomes of patients with pituitary adenoma. The purpose of this study is to report the incidence, clinical presentation and postoperative complications, and to identify possible risk factors for PA based on the data from our center.

## Methods

### Patient population

The Huashan Hospital (Shanghai) is one of the largest pituitary tumor centers in China. The study population included all pituitary adenoma patients presenting at this center from 3rd January 2005 to 31st December 31 2007 who underwent surgical therapy for pituitary adenoma by transsphenoidal microsurgery or craniotomy. A total of 2021 patients aged from 7 to 88 years old (mean, 44.5±14.4 year) were recruited. All patients were informed about the purpose of the study and signed a written consent form. The study was approved by the institutional review board of Huashan Hospital, Fudan University.

### Data collection

A structured database was specifically designed to collect data on basic socio-demographic information, endocrine function tests, clinical manifestations, imaging results, pathological staining, postoperative complications and medical history. Relevant data were also collected from inpatient medical records, including medical treatment information (adherence, dosage, adverse reactions and drug interactions).

### Cases and controls

Patients with PA were compared with the remaining non-PA patients with pituitary adenoma during the study period. A matched case-control study was conducted to analyze risk factors for PA and clinical characteristics of PA. Cases were all patients with PA. Using a computer-generated random selection scheme, controls were randomly selected from the study population. Controls were matched (cases to controls ratio: 1:2) to cases by sex, tumor type, size of tumor and age(see S1 Annex).

### Diagnostic criteria

Patients who met 2 or 3 of the criteria of the following were diagnosed with PA:

1. Typical clinical presentation of PA (such as sudden headache, vomiting, visual impairment and meningism.
2. Signs of hemorrhage on imaging.
3. Pathological diagnosis of hemorrhage.

Hypertension was defined as taking medication for hypertension or having either a systolic blood pressure ≥140 mm Hg or a diastolic blood pressure ≥90 mm Hg [15]. The 1999 World Health Organization diagnostic criteria were used to diagnose diabetes [16]. Adenomas that were 10 mm or larger were defined as macroadenomas, and those less than 10 mm in diameter were regarded as microadenomas.

### Imaging

All patients underwent computed tomography (CT) scan and/or magnetic resonance imaging (MRI). Plain-contrast CT scan determined the dimension, form, and extension of the adenoma in the coronal plane. MRI was performed to determine the general position of the adenoma and extension to the surrounding structures in the coronal, sagittal, and frontal planes. CT scans and/or MRI were also used to evaluate tumor size and the characteristics of the PA.

### Histology

Tumor types were determined by standard immunohistochemical staining at the department of Neuropathology, Huashan Hospital[14].

### Statistical methods

Pearson's chi-squared test, Pearson's statistic with a continuity correction, and bilateral Fisher’s exact test, as appropriate, were used to compare the frequency distribution of categorical variables between groups. To assess risk factors for PA, logistic regression analyses adjusting for confounders were conducted with the whole group. Univariate and adjusted odds ratios (OR) and the corresponding 95% confidence intervals (CI) were calculated. All data were analyzed using the Statistical Package for Social Sciences (SPSS), version 10.0. Two tailed P-values less than 0.05 were considered to be statistically significant.

## Results

**Fig 1:**
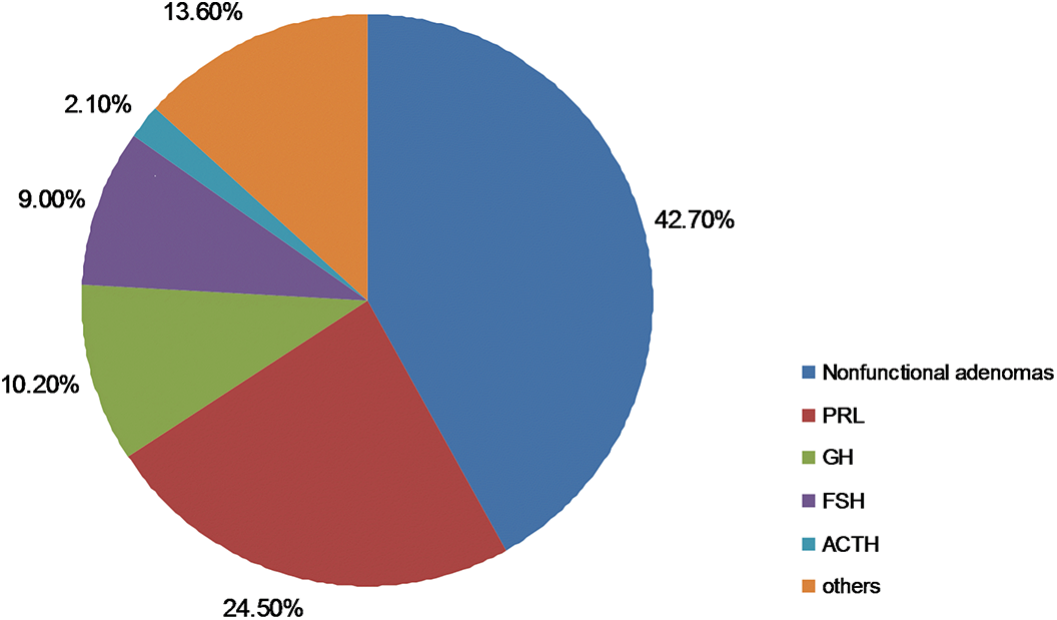
The subtypes of pituitary adenoma in 2021 patients. PRL, prolactin; GH, growth hormone; LH, luteinizing hormone; ACTH, adrenocorticotropic hormone; TSH, thyroid-stimulating hormone; FSH, follicle-stimulating hormone. Others include multi-hormonal, LH, TSH, and unknown type.

2021 patients (mean age: 44.5±14.4y) with pituitary adenoma confirmed by pathology were recruited into this study. The clinical characteristics of all patients are shown in Table 1. The tumor subtypes based on immunohistochemical staining results are shown in Fig 1, with 45.5% and 54.5% patients being diagnosed with macroadenoma and microadenoma respectively.

**Table 1:** Characteristics of all 2021 patients with pituitary tumors, 2005–2007.

Characteristics		n	%
**Age**			
	**≤35**	586	29.0
	**36~45**	519	25.7
	**46~55**	422	20.9
	**≥56**	494	24.4
**Gender**			
	**Female**	1076	53.2
	**Male**	945	46.8
**Incidental adenoma**		92	4.6
**Second surgery**		168	8.3
**Diabetes mellitus**		45	2.2
**Hypertension**		139	6.9
**PA**		97	4.8
**Tumor size**			
	**Microadenoma**	1101	54.5
	**Macroadenoma**	920	45.5

### Incidence of pituitary apoplexy

97 (4.8%) patients were diagnosed with PA. The mean age of patients with PA was 50.1±13.9y. Table 2 shows the incidence of PA in different subgroups. There was a significant difference in the proportion of patients with apoplexy at different ages (p = 0.000; Table 1). PA occurred significantly more commonly in men (7.41%) than in women (2.51%). The incidence of PA was 10.11% in patients with macroadenoma and only 0.36% in patients with microadenoma. Most patients with PA (95.6%) were found to have macroadenoma.

**Table 2:** Incidence of PA in subgroups.

Characteristics		PA (n = 97)	%	P
**Age (years)**				
	**≤35**	16	2.73	0.000*
	**36~45**	18	3.47	
	**46~55**	28	6.64	
	**≥56**	35	7.09	
**Women**		27	2.51	0.000*
**Men**		70	7.41	
**Hypertension**		9	9.28	0.338*
**Diabetes**		2	2.06	1.000
**History of pituitary operation**		3	1.79	0.056*
**Pathological staining**				
	**Null (non functional)**	63	7.31	NA
	**Functional**	34	2.93	
	**PRL**	7	1.41	
	**GH**	8	3.88	
	**ACTH**	1	2.33	
	**TSH**	2	7.41	
	**FSH**	10	5.49	
	**LH**	0	0	
	**Others**	4	3.4	
	**Multiple staining**	2	2.3	
**Total patients**				
	**Macroadenoma**	93	10.11	0.000*
	**Microadenoma**	4	0.36	
**Men**				
	**Macroadenoma**	67	13.67	0.000*
	**Microadenoma**	3	0.66	
**Women**				
	**Macroadenoma**	26	6.05	0.000*
	**Microadenoma**	1	0.15	

NA, not applicable; PRL, prolactin; GH, growth hormone; LH, luteinizing hormone; ACTH, adrenocorticotropic hormone; TSH, thyroid-stimulating hormone; FSH, follicle-stimulating hormone. PA, clinical pituitary apoplexy.

* Pearson's chi-squared test.

In the subgroup of patients with macroadenoma, the incidence of PA was 13.67% in men and 6.05% in women. In the subgroup of patients with microadenoma, the incidence of PA was 0.66% in men and 0.15% in women.

### Clinical presentation

Table 3 illustrates the comparison of clinical presentation between patients with PA (n = 97) and the controls (n = 194). The top three presenting symptoms in PA were headache, visual deterioration, and vomiting. Visual deterioration, headache, and decreased libido were more frequent in the control group.

**Table 3:** Symptoms at presentation.

	PA (n = 97)		Non-PA (n = 194)		P
**Symptom/sign**	n	%	N	%	
**Headache**	84	86.60	64	33.00	0.000*
**Visual deterioration**	60	61.86	126	64.95	0.605*
**Vomiting**	39	40.21	3	1.55	0.000*
**Ptosis**	25	25.77	0	0.000	0.000*
**Decreased libido** ^**¶**^	7	10.00	41	28.87	0.000*
**Head trauma**	7	7.21	21	10.82	0.325*
**Diplopia**	6	6.18	1	0.52	0.004^‡^
**Acromegalic appearance**	6	6.19	18	9.29	0.366*
**Fever**	5	5.15	1	0.52	0.009^†^
**Blindness**	5	5.15	1	0.52	0.009^†^
**Cushingoid appearance**	5	5.15	2	1.03	0.079^†^
**Amenorrhea** ^**§**^	4	14.81	7	8.54	1.000^†^
**Hypothyroidism**	2	2.06	18	19.15	0.022*
**Altered menstrual cycle** ^**§**^	1	3.70	14	26.92	0.006*
**Galactorrhoea** ^**§**^	1	3.70	9	17.31	0.060^†^
**Cold intolerance**	1	1.03	13	6.70	0.066^†^
**Gynaecomastia** ^**¶**^	0	0	1	0.70	—

* Pearson's chi-squared test.

† Pearson's statistic with a continuity correction.

‡ Statistical analysis done by Fisher’s exact test.

§ These calculations include only women.

¶ These calculations include only men.

### Risk factors for pituitary apoplexy

In a univariate analysis, odds ratios for PA increased in patients older than 45 years old. PA was more frequent in those aged 46~55 (OR = 2.53 95%CI 1.35~4.74) and in the >56 years old group (OR = 2.71 95%CI 1.48~4.97) than in the control group (ages≤ 35 years). But in multiple logistic regression models, age was not significantly associated with PA (P>0.05).

In comparison with women, men were associated with a higher odd ratio of developing PA (OR: 2.54, 95% CI: 1.59~4.07). The positive pathological staining group, including PRL, FSH, GH, ACTH, TSH, and multiple staining, pituitary tumors, was associated with an increased odds ratio of PA compared with negatively pathological staining tumors (OR: 2.04, 95% CI:1.29~3.23). Patients with macroadenoma had significantly higher incidence of PA than those with microadenoma (OR: 26.46, 95% CI: 9.66~72.46). A history of diabetes mellitus, hypertension, and tumor recurrence were not significantly associated with PA (Table 4).

**Table 4:** Predictors of clinical PA in pituitary adenoma patients.

		Univariate		Adjusted	
Factors		OR (95% CI)	P	OR (95% CI)	P
**Age (years)**					
	**≤35** *	1		1	
	**36~45**	1.28(0.65~2.54)	0.479	0.92(0.45~1.88)	0.827
	**46~55**	2.53(1.35~4.74)	0.004	1.50(0.77~2.92)	0.234
	**≥56**	2.71(1.48~4.97)	0.001	1.52(0.78~2.93)	0.217
**Gender**					
	**Female** *	1		1	
	**Male**	3.11(1.98~4.90)	0.000	2.54(1.59~4.07)	0.000
**Diabetes mellitus**		1.41(0.69~2.87)	0.341	0.55(0.12~2.52)	0.443
**Hypertension**		0.92(0.22~3.86)	0.910	0.92(0.42~1.99)	0.828
**Tumor recurrence**		0.34(0.10~1.09)	0.074	0.34(0.10~1.11)	0.074
**Types of pathological staining**					
	**Positive staining** * ^**†**^	1		1	
	**Negative staining** ^**¶**^	2.61(1.73~3.99)	0.000	2.04(1.29~3.23)	0.002
**Adenoma size**					
	**Microadenoma** *	1		1	
	**Macroadenoma**	30.84(11.29~84.25)	0.000	26.46(9.66~72.46)	0.000

Crude OR, crude odds ratios; Adjusted OR, adjusted odds ratios; CI, confidence interval.

* reference

† including PRL, FSH, GH, ACTH, TSH, and multiple staining

¶ non-functioning tumor type

### Postoperative complications

Table 5 shows a comparison of postoperative complications between patients with PA (n = 97) and controls (n = 194). Temporary diabetes insipidus was the most frequent postoperative complication in both PA and control groups.

**Table 5:** Surgical approach and postoperative complications.

		PA(n = 97)		non-PA(n = 194)		P
		n	%	N	%	
**Surgical approach**						
	**Transsphenoidal**	93	95.88	166	85.57	0.010*
	**Transcranial**	4	4.12	27	13.92	
**Surgical Resection**						
	**Total resection**	80	82.47	163	84.46	0.690*
	**Subtotal resection**	12	12.37	24	12.44	
	**Partial resection**	5	5.15	6	3.11	
**Complications(total)**		74	76.29	159	81.96	
	**Electrolyte disturbance**	18	18.56	13	6.70	0.002*
	**Diabetes insipidus**	69	71.13	157	80.93	0.059*
	**Postoperative hemorrhage**	1	1.03	5	2.58	—
	**Postoperative infection**	1	1.03	3	1.55	—
	**Cerebrospinal rhinorrhea**	2	2.06	2	1.03	—
	**Death**	1	1.03	2	1.03	—

PA, clinical pituitary apoplexy.

* Pearson Chi-Square test.

## Discussion

PA is an acute syndrome characterized by visual impairment, vomiting, headache, and decreased consciousness caused by hemorrhage and/or infarction of the pituitary gland [1]. It is a challenge for doctors to diagnose and manage patients with pituitary apoplexy. Unfortunately, no clinical controlled trials have yet been undertaken to guide management of pituitary apoplexy to optimize treatment.

Diagnostic criteria for PA vary between studies, and the differential diagnosis of PA includes many common neurological emergencies. PA is often the first presentation of an unknown pituitary tumor [5,17]. It is probably the above-mentioned factors that can lead to diagnostic delays and difficulties. Clinical assessment and typical medical imaging have been found useful for confirming the diagnosis of PA.

In this study, PA was defined on the basis of clinical signs coupled with imaging or histopathological evidence. The definition of PA was similar to some recently published studies [11,12]. The most common clinical features in this study were headache, nausea, vomiting, and visual deterioration. These findings correspond with previous studies [2,4,6,8].

Clinical presentation can be either subacute or acute, which is largely determined by the extent of haemorrhage around the saddle area and a sudden increase in intracranial pressure. A diagnosis of PA should be considered in patients presenting with this clinical presentation, and assessment of visual fields must be undertaken. Headache is often the most consistent, frequent, and earliest symptom of PA. Headache is commonly accompanied by nausea and vomiting. The rapid increase in the intrasellar contents and intrasellar pressure usually presents as sudden onset of headache. Visual deterioration has been reported in 52–90% of patients with PA. In some studies male sex was more predisposed to visual deterioration following PA [18,19].

A review of previous studies of PA found an incidence rate varying from 1.6% to 21%. In the current study, 4.8% (97 in 2021) patients operated on for pituitary adenomas were diagnosed with PA. This variation in incidence of PA may be related to differences in diagnostic criteria. A recent study by Cinar et al [20] indentified thirty-two patients (5.8%) with clinical and 81 patients (14.8%) with subclinical PA. Besides the differences in diagnostic criteria used in the studies, differences between study populations in gender, age, size, pathological type, and availability of emergency neurosurgery service should also be considered as important factors contributing to the extreme variability in the reported incidence of PA. For example, 10.11% of patients with macroadenoma were identified as PA in our series, while 10.6% of microadenomas were reported as PA by Möller-Goede with a total prevalence of PA of 7.3% [11]. Wakai S et al [21] found that the incidence of PA was correlated with age. PA incidence also increased with age in our study, and patients with PA have a higher mean age than those who are non-PA (50.1±13.9y vs 44.5±14.4y). But multiple logistic regression analysis shows that age is not an independent risk factor for PA. In addition, the mean age of PA patients in our study was higher than the mean age of PA patients in other series [2,20].

PA is correlated with sex in our results. Of patients with PA, 72.2% were men and 27.8% were women. PA occurred three times more often in men (7.41%) than in women (2.51%), which is similar to the difference reported by Liu [22] and Möller-Goede [11]. Some studies did not find any correlation between PA and sex [20,21]. The higher incidence in men might be related to a high rate of macroadenoma in men, but multivariate and univariate analyses show that male sex is an independent risk factor for PA with an odds ratio of 2.82 (95% CI: 1.63~4.86). This may be because men are more likely to have poor cardiovascular function with increasing age. Hemorrhage or infarction of the pituitary gland is more likely in men.

PA is expected in patients with pituitary macroadenoma but it does also occur in patients with microadenoma, as reported by Randall [23] and confirmed in our report. Patients with macroadenoma have a significantly higher incidence of PA (OR: 26.46, 95% CI: 9.66~72.46) than their counterparts with microadenoma. Hence the size of the adenoma remains a major risk factor for PA. Macroadenoma may be more invasive and hemorrhage more easily. An important consideration is that bleeding affects measurement of pituitary tumor size.

Cinar et al found that cavernous sinus invasion is a risk factor for apoplexy [20]. Cavernous sinus invasion has not been investigated in our study, but it is well known that cavernous sinus invasion is more prevalent in patients with macroadenoma than microadenoma. We hypothesize that tumor invasion and tumor size interact with each other when PA occurs. Of note, the classification system for PA was developed for a group of macroadenomas and does not include microadenoma invasion of the sella dura or the cavernous sinus [24].

In agreement with some previously published studies [20,25], the incidence of PA is significantly higher in patients with clinically non-functioning tumors. For example, Möller-Goede et al [11] reported that 76% of patients with non-functioning tumors had PA. But Mou et al [12] found that incidence of apoplexy was significantly higher in patients with functional pituitary tumors than in patients with non-functional tumors. In our study, 64.9% of patients with PA had non-functioning tumors. The incidence of PA in patients with non-functioning pituitary adenomas is 7.31%, which is approximately 2.5 times higher than those with functional adenomas (2.93%). The higher incidence of PA in nonfunctional adenoma might be related to a higher percentage of macroadenoma, but multivariate and univariate analyses show that non-functioning tumor is an independent risk factor for PA with an odds ratio of 2.04 (95% CI 1.29~3.23).

Because of degenerative changes of microvasculature in the pituitary gland, diabetes and hypertension are thought to correlate with PA. In our study, however, history of hypertension and diabetes mellitus history were not risk factors for PA. In agreement with Biousse et al [26] [20], there is no evidence that diabetes or hypertension is more common in patients with PA.

The detailed pathophysiological mechanisms involved in the genesis of pituitary tumor apoplexy are not clear. It is uncertain whether the pathological process is a primary hemorrhage or whether the event is really a hemorrhagic infarction. The pathophysiology of the clinical manifestations of pituitary tumor apoplexy can be divided into any combination of the following mechanisms: sudden increase in intrasellar pressure, sudden increase in intrasellar contents, and leakage of blood or necrotic tissue into the subarachnoid space[25].

Both hemorrhage and infarction can lead to occlusion of the internal carotid artery and subsequent cerebral ischemia. In PA, there are two possible mechanisms of cerebral artery occlusion: vasospasm or a mass effect compressing the artery, both of which have been reported in the literature[27].

The nature of pituitary tumors is closely related to their vascularization. McCabe et al [28] found high vascular endothelial growth factor (VEGF) mRNA expression in non-functioning tumors compared with other types of pituitary tumors and normal pituitaries [28]. Anti-VEGF antibody has inhibitory effects on pituitary tumor genesis in PRLoma models [29].

It is clear from the existing literature that PA is associated with high mortality, but only one death occurred after surgery in the PA group of our study. Nielsen found that survival is independent of the occurrence of PA in operated patients with nonfunctioning adenoma [9].

Regarding postoperative complications, temporary diabetes insipidus was the most common complication in both PA (71.13%) and control groups (80.93%). Incidence of electrolyte disturbance was significantly higher in PA patients than in the non-PA control group (18.56% vs. 6.70%, P = 0.002). No differences were found between the two subgroups for other postoperative complications. Incidence of these postoperative complications in PA was similar to a previous report in 4050 patients of pituitary adenomas in our centre [13].

The strengths of our study are the large number of patients, and logistic regression analysis to investigate risk factors for PA. This is superior to univariate analyses performed by others. although some risk factors, such as anticoagulant use and immunohistochemical analysis, are not included in our study. Cinar et al [20] found that Ki-67 labeling index, p53 positivity, antithrombotic therapy, and somatostatin analogue do not predispose to PA.

Ours is a new database for collecting clinical, biochemical, radiological and clinical outcome data specifically focused on PA in China. Our study found that PA is not a rare event. Different from previous reports, we found that age, diabetes mellitus and hypertension are not risk factors for PA. These study data provide a complete description of patients with PA. The relatively large number of cases recorded in our database may be used to further evaluate diagnosis and treatment.

We conclude that PA occurs more frequently than previously assumed. The properties of the tumor itself (macroadenoma and non-functioning pituitary adenoma) are risk factors for PA. There is also a sex difference, and male sex is an additional risk factor for PA. We find no significant difference in the overall incidence of complications in patients who have PA compared with non-PA patients. These risk factors may help earlier diagnosis of pituitary apoplexy in patients with pituitary adenoma.

## Supporting Information

S1 Annex Data on comparison of complications and symptoms between patients with pa and controls. (EXCEL)
(XLS)
